# **Electronic Dose Monitoring Feedback (EMF) with Youth Living with HIV: Qualitative Exploration of Reactions to Viewing Dosing Calendars**

**DOI:** 10.1007/s10461-025-04820-w

**Published:** 2025-07-21

**Authors:** Eamonn McGonigle, Megan Mueller Johnson, Heather Tucker, Michael Hudgens, Ronald Dallas, Keith J. Horvath, Rachel Goolsby, Elizabeth Secord, Murli Purswani, Daniel Reirden, Mobeen Rathore, Lisa-Gaye Robinson, Aditya H. Gaur, K. Rivet Amico, TERA Study Team

**Affiliations:** 1 University of Michigan, School of Public Health, Department of Health Behavior and Health Equity, Ann Arbor, MI US; 2 University of Washington, School of Public Health, Department of Global Health, Seattle, WA US; 3 University of North Carolina at Chapel Hill, Gillings School of Public Health, Department of Biostatistics, Collaborative Studies Coordinating Center, Chapel Hill, NC US; 4 St. Jude Children’s Research Hospital, Department of Infectious Diseases, Memphis, TN US; 5 San Diego State University, Department of Clinical Psychology, San Diego, CA US; 6 Wayne State University, Detroit, MI US; 7 BronxCare Health System, Bronx, NY US; 8 University of Colorado Denver, Children’s Hospital Colorado, Denver, CO US; 9 University of Florida Center for HIV/AIDS Research, Education and Service (UF CARES), Jacksonville, FL US; 10 South Florida CDTC, Ft. Lauderdale, FL US

**Keywords:** YLWH, Viral Suppression, Intervention, mHealth, Coaching, EDM, Youth

## Abstract

Electronic dose monitoring with feedback (EMF) offers an opportunity to visualize daily dosing behaviors that can otherwise be difficult to appreciate. Visual displays, including early, on-time, late, or missed doses over time, can foster insights around patterns of dosing. Reactions to seeing these patterns among youth with HIV (YWH) who struggle with adherence are important to consider with EMF. YWH (ages 14–25) participating in the ATN152 TERA intervention were presented withsmart-bottle-generated 1-month EMF calendars as part of their sessions with a remote ‘coach’. To characterize how youth reacted to these visualizations, transcripts from this portion of the coaching sessions were thematically analyzed. A total of 64 youth-coach discussions were characterized across 37 unique participants (22 years old on average, 81% Black/African American, 46% acquired HIV vertically). Six main reactions to EMF calendars were identified: feelings of pride/satisfaction, empowerment/motivation, positive surprise, negative surprise, shame/guilt, and/or neutral reactions. Although most reactions were positive, those with negative reactions tended to be in response to calendars showing low adherence. Over a quarter (28%) of youth ranked the EMF as one of the most beneficial/helpful aspects of the intervention when asked about experiences with intervention tools within the coaching session. A calendar EMF appeared to offer unique opportunities to explore adherence in the context of a supportive counseling session.

## Introduction

In the past 30 years, significant advances in antiretroviral therapies (ART) have dramatically extended life expectancies of people with HIV (PWH) [1, 2]. New options for HIV treatment include long-acting injectable therapy, presently available in the US for virally suppressed individuals [3, 4]. For those who struggle with adherence to oral ART, who are not virally suppressed [5], innovative strategies to promote and support daily dosing of oral ART are still needed. Low rates of viral suppression among youth with HIV (YWH)-- only 36% of those engaged in HIV care and prescribed ART in one recent large study in the US [6]-- suggest substantial and potentially unique adherence challenges for youth. Compared to adults, youth and adolescents face unique barriers to adherence related to cognitive (e.g., executive functioning, emotional regulation) and identity development throughout this period of time, in addition to challenges with housing, stigma, memory, healthcare access, and income [7–10]. Also, intersectional stigma [11] caused by structural racism plays a significant role in adherence [12, 13], important to consider for Black youth, who were the majority of the research participants in this study.

Leveraging mobile health technologies to promote ART adherence, particularly those connected to cell-phone usage among adolescents in the US, has received considerable attention [14, 15]. Such methods to promote adherence may be helpful to assist with addressing barriers to adherence for youth [16–18], and more specifically for Black youth [19]. For example, electronic adherence and dose monitoring (EDM) devices tethered to outreach via cell-phone text-messages offer unique opportunities to engage youth in real-time interventions. EDM (e.g., smart pill cases and smart bottles) generated adherence estimates appear to accurately measure adherence more than self-report or pharmacy-based measures [20] and have been used in a number of chronic health conditions [21–23]. EDMs store medication and register “opening events,” which are inferred to mean taking a dose of the medication. Devices that signal opening events transmitted through cellular technologies to a server can be used to “monitor” dosing in real or near-real time, depending on whether the device is in an area within the cellular network.

EDM data can be consolidated into visual displays of granular daily dosing across time, creating opportunities to share information creating opportunities to provide personalized adherence feedback to patients. Research with adults has suggested personalized graphic displays in a calendar format have particular appeal for ease of understanding [24]. Calendar electronic dose monitoring feedback (EMF) offers objective data on day-to-day adherence patterns instead of adherence in general or in aggregate, which can be particularly useful for daily, repeated behaviors. Little data has been presented to date to advise the process of feeding back adherence calendars with YWH who struggle with adherence. Reactions from youth to EMF visualizations may offer valuable insights into this process. Viewing calendars where each day is characterized with a visual indication of dosing outcome (on-time, late, or missed) can highlight patterns of dosing and adherence gaps but could also produce negative or defensive reactions among YWH, particularly for those struggling with adherence.

Data from the Triggered Escalating Real-Time Adherence (TERA) [25, 26] study was used to gather insights into youth reactions to viewing their dosing-calendar EMF. TERA evaluated a 3-session, 12-week virtual coaching intervention among YLWH living in the US within the Adolescent MedicineTrials Network for HIV Interventions (ATN). The study and main outcomes are presented elsewhere [25, 26]. Briefly, results suggested that youth assigned to the intervention condition had significantly better EDM-based adherence but were not different in viral suppression at the primary endpoint [26]. Here we present findings from a qualitative exploration of coaching session transcripts to gain insights around youth reactions to viewing their EMF-calendars to prepare for future EMF-based interventions.

## Methods

### Participants

 Participants were, per protocol [25, 26], between 14 and 25 years old, had viral load at baseline of 200 copies/mL or higher, and were on an ART regimen that was dosed once a day. Demographics for the full TERA study are presented elsewhere [25, 26]. Participants were recruited from eight clinical research sites (Colorado, Florida, Georgia, Maryland, Michigan, New York, and Tennessee). Youth (13 to 24 years of age) had to have a documented HIV-1 RNA plasma ≥ 200 copies/mL (within 45 days of enrollment), on an antiretroviral therapy regimen (dosed once a day) for at least 24 weeks, aware of HIV status, and have a cellular phone. Youth with severe cognitive or mental health challenges that would interfere with ability to participate, pregnant at time of enrollment, already enrolled in an adherence study, or already using an electronic monitoring device were not eligible to participate. All data for the current evaluation of coaching sessions were collected between April 2018 and December 2019.

### Procedures

**Fig. 1:**
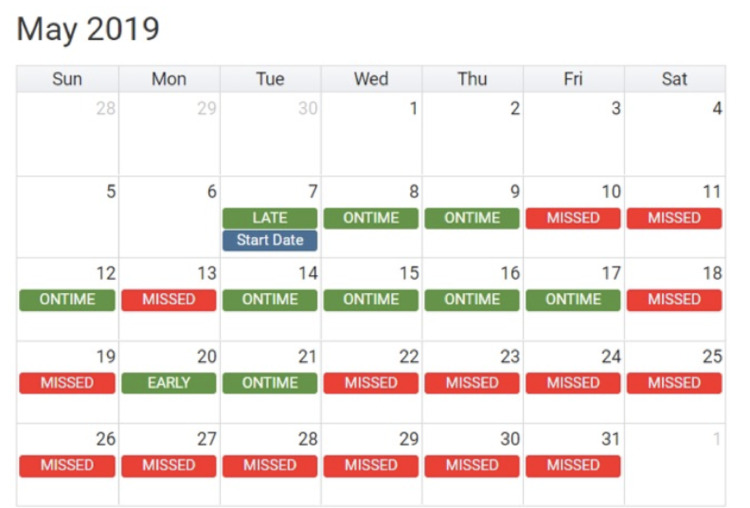
Example of the calendar EMF in the TERA Intervention

 As presented elsewhere [25, 26], youth were given an AdhereTechⒸ EDM bottle to monitor daily dosing and generate a dosing calendar. Every time the bottle was opened, data were collected on day and time, which was interpreted to signal taking a dose of medication. As part of the TERA intervention, delayed dosing (within 2-hours of scheduled dose time with no device opening) prompted texted follow-up (automated followed by coach-generated outreach). EDM data was used to create an EMF calendar (Fig. 1) for participants to view with their coach as part of their coaching sessions at study weeks 4 (the 2nd coaching session) and 12 (the 3rd and final coaching session). The calendar displayed each day with “ON TIME,” “EARLY,” or “LATE” in green or “MISSED” in red. During the audio-recorded coaching sessions, participants were presented with their EMF dosing calendar and coaches started with a general question about reactions to the calendar. Coaches then drew from Motivational Interviewing [27] and Next Step Counseling [28, 29] to help youth identify strengths and areas for improvement, ultimately concluding with youth-identified adherence related goal(s). Other parts of the coaching sessions included discussion of day-to-day life and goals, experiences related to living with HIV, and their feelings about the TERA study.

Data were extracted from de-identified transcripts of virtual (video-conferenced) coaching sessions at study-weeks 4 (the 2nd coaching session) and 12 (the 3rd and final coaching session). The portion of the session where the EMF calendar was presented and discussed was extracted for analysis. Additionally, at the week 12 session, coaches explored experiences with the intervention as part of intervention termination processing and planning for sustaining or improving adherence. Coaches listed out six core elements of the TERA intervention (tools [bottle features and text messages], coach support, family/friend support, clinical support, personal strength, and EMF calendars) and asked participants to reflect on the components they found most helpful. These discussions were also extracted from the deidentified session transcripts for review.

### Analyses

 Transcripts were analyzed using inductive thematic analyses. After familiarization with the data, a codebook was created and updated as and when needed during code application in Dedoose [30]. A total of 10% of transcript excerpts were randomly selected and reviewed by a second team member for consistency of code application, achieving 90% agreement, with any discrepancies resolved through discussion and codebook updated as needed. Themes were also quantified to characterize the percentage of participants whose discussions with their coach included a given theme. Results were integrated into a visual display summarizing themes and definitions, percent of participants contributing to the theme and example quotes for the week-4 and week-12 coaching sessions.

### Compliance with Ethical Standards

Study procedures for the TERA intervention study were reviewed and approved by a central IRB (the ATN Coordinating Center at University of North Carolina, Chapel Hill) and monitored twice a year by the ATN’s Safety Monitoring Committee (SMC). All participants provided informed consent prior to participation.

### Data Availability

TERA data are available through the NICHD Data and Specimen Hub (https://dash.nichd.nih.gov/).

## Results

The 37 intervention-arm participants generated 72 coach-participant transcripts (36 for the first follow-up session at week 4 and 36 for the second follow-up session at week 12^1^). Participants were on average 22 years of age (*SD* 2.8, range of 14 to 25 years old). The vast majority identified as African American or Black (81%), half (51%) identified as Female, and 11% identified as Latino/a. Nearly half (49%) were employed and over half (58%) reported Bisexual, Gay, or Lesbian sexual identity. Slightly over half (54%) reported horizontal mode of HIV acquisition (horizonal transmission: HT), with the remainer (46%) reporting vertical (vertical transmission: VT).

### Main Themes

**Fig. 2:**
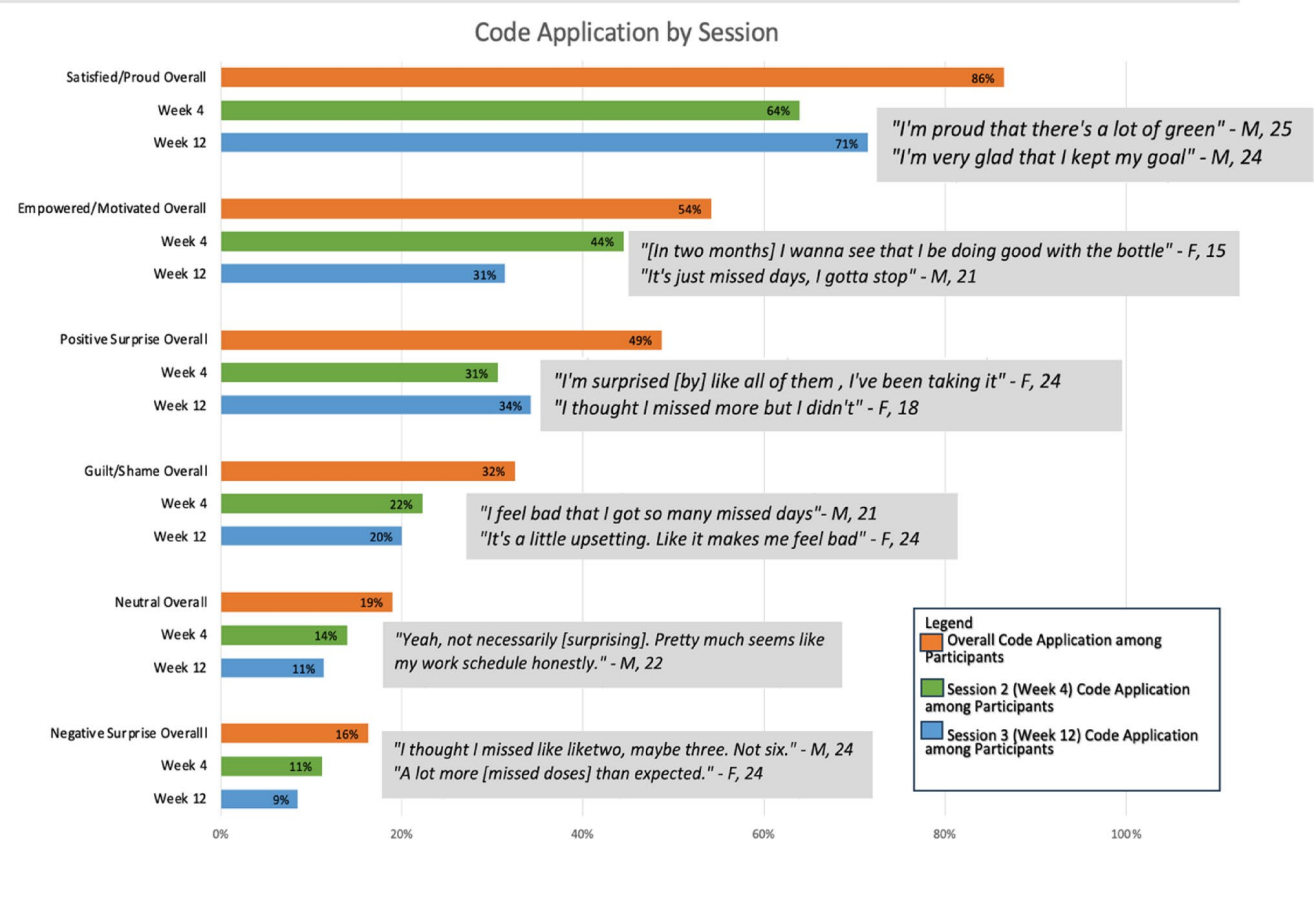
Visual Display of Themes in Reactions to Calendar EMFs During Coaching Sessions, percent of participants with the theme (ever, *N* = 37) and at session 2 (week 4 on study, *N* = 36) and at session 3 (week 12 on study, *N* = 36) and example quotes. There were 3 coaching sessions in TERA. Session 2 (at week 4 on study) and Session 3 (at week 12 on study) included the review of the participant’s EMF calendar. M Male identifying; F Female identifying; Age.

 Across all transcripts, six unique, non-mutually exclusive themes emerged (defined in Table 1). Figure 2 summarizes themes overall and by coaching session. These themes ranged in positive and negative affective response. Feeling satisfied or proud was the most frequent single code with participants expressing sentiments such as “It’s like every day. I’m proud of myself” (F, 22, 89% Adh, VT). By session, at week 4, 64% of participants expressed satisfaction or pride, while 69% expressed this in the week 12 session. As presented in Fig. 2, this discourse is reflected by participant verbalizations of a sense of accomplishment and success. The next most common response was a feeling of surprise, positive or negative. About half of the participants shared some aspect of being surprised by the data presented in the calendars. These reactions included quotes such as “I thought there was going to be a lot more early [doses], but I’m glad it’s on time” (F, 18, 100% Adh, VT) and “I didn’t think I was gonna be like on time and like take it.” (F, 18, 91% Adh, VT) to quotes such as “it’s different [than what I expected] because I didn’t expect to see more reds (missed doses)” (M, 20, 29%, HT). Positive surprise was present for 31% of the youth in the week 4 coaching session and 33% in the week 12 session. Negative surprise was less common, with 11% of the sessions for week 4 and 8% in the week 12 sessions sharing a reaction coded for this.

**Table 1:** Themes and definsition

Theme	Description and example quotes
Feeling of Pride/Satisfaction	Participants described feeling proud of their dosing results. They are satisfied with the calendar and happy with themselves. Continue with their current adherence plan due to a satisfactory calendar.
Feeling of Positive Surprise	The reaction of surprise was positive, and the results of the dosing data were better than expected.
Feeling of Negative Surprise	The reaction of surprise was negative, and the results of the dosing data were worse than expected.
Feeling of Empowerment or Motivation	Dosing data results either encouraged them to improve or set goals for their drug adherence due to an unsatisfactory calendar or continue with their current adherence plan due to a satisfactory calendar.
Feeling of Guilt or Shame	Participants felt shameful or guilty over the results from their dosing data calendar. They are unsatisfied and/or upset with how the results turned out.
Neutral Reaction	Transcript text did not suggest a particular emotion or reaction in response to being presented with the dosing data results. Results were unexciting, as expected, or uninteresting

Participants also discussed a sense of motivation and empowerment seeing their dosing data (44% in the week 4 session and 31% in the week 12 session). This was different from the code for pride/satisfaction as those who felt motivated and empowered had varying adherence levels and emphasized a behavior change alongside reflections on dosing. These included quotes such as “I wanna say I can do less misses. I could, I got confidence in myself. I think I could do it.” (F, 22, 89% Adh, VT) and “Yes I need the whole thing to be green. When I look at it next time, I need it green.” (M, 23, 20% Adh, HT). Shame or guilt was identified for 22% of participants in week 4 and 19% in week 12 sessions; however, 67% of those expressing shame or guilt were doing so in reaction to dosing calendars characterized by less than 80% adherence. Finally, 14% and 11% of participants in week 4 and week 12 expressed neutral reactions to viewing their calendars. Overall, reactions were dominated by positive responses in comparison to negative or neutral themes.

When the ranking of TERA components was done at the end of the third coaching session at week 12, participants ranked tools (EDM bottle and real-time text messaging) as the most helpful component (83%), with coaching support coming second (69%) and EMF adherence calendars coming third (28%).

## Discussion

Our evaluation of coaching session transcripts where youth were presented with their EMF calendar as part of a supportive counseling session suggested largely favorable reactions by YWH. Negative reactions were present but uncommon. The main themes we identified from transcripts of the intervention coaching sessions when youth were presented with their EMF calendar (in order of frequency) included feeling proud, surprised in a positive way, motivated or empowered, guilt or shame, neutral, and surprised in a negative way. The week 4 session (the first-time youth saw their EMF calendar) and the week 12 session appeared generally similar in themes. Youth also ranked seeing their EMF calendars among the top 3 aspects of the intervention they found helpful.

One potential advantage of presenting daily behavior in a visual format like a calendar is that it may offer a unique perspective that is not otherwise available. Expressing surprise was fairly common among YWH, both when seeing their dosing calendars for the first time and when seeing it again about 2 months later. We hypothesize that the calendar EMF gave youth an opportunity to reflect on adherence in a highly nuanced, temporal way that otherwise would be too difficult to reproduce using recall alone. YWH have higher rates of HIV-associated neurocognitive disorders which can challenge adherence directly, and limit reflecting on past adherence behavior. In addition to interventions targeting improvements in neurocognitive resources [31], strategies that can visually highlight patterns in adherence can draw attention to adherence as a non-static dynamic process [32]. Although the potential impact of this “new information” was not characterized in our research, the impact of activating information of this nature is consistent with social behavioral models of adherence that identify accurate information as influential [33, 34]. 

A critique of interventions relying too heavily on the monitoring and feedback of these data points is that the EMF visualization risks adopting an incomplete picture of events that have a multitude of situational characteristics [35]. Monitoring of dosing favors time over other aspects of the situations surrounding dosing and non-dosing. Our exploration of youth reactions to an EMF calendar was in a very specific context- these calendars were reviewed with a coach in a supportive counseling session. The TERA intervention included a number of person-centered activities with the incorporation of Motivational Interviewing strategies and principles. We cannot speak to whether or not youth would have experienced EMF calendars positively if presented outside of this supportive relationship or outside of a relationship entirely (e.g., as a product available from an app untethered to an interpersonal exploration of it). The participants experiencing guilt or shame, or negative surprise were supported by the coach. Per protocol, all participants were first asked what *they* made of the calendar. Coaches then worked with participants to identify patterns of success and areas for improvement. Monitoring apps should consider the potential role of coaches or human interface when using EMF, and research is needed to determine whether reactions to EMF calendars are similar when data is provided electronically, as part of an app, without coach assistance in processing the feedback.

Several limitations in the current work warrant consideration. In addition to a very specific scope of generalizability (e.g., EMF reactions were collected in the context of a coaching intervention session), the use of session transcripts for secondary exploration has limitations. We cannot speak definitively to what participants felt when seeing their dosing calendar; rather, we can only characterize the discourse around viewing them. Participants may have shared only positive reactions with their coach despite feeling negatively. They may have presented themselves in a way they thought the coach would appreciate. Similarly, when discussing aspects of the TERA intervention they found helpful, a strong social desirability bias may have led participants to identify the coach (the person asking the question) as most helpful. The extent to which this “in-session” context may have influenced participant-coach interactions is somewhat challenged by the presence of negative reactions and the ranking of other intervention components (besides the coach) as helpful. Nonetheless, careful future research specifically focused on how youth respond to EMF dosing calendars, as a self-reviewed tool or as a part of a guided exploration, is needed. Coaches were trained in communication and counseling strategies that allowed for exploration of experiences around “successful” adherence and well as days or periods of days without dosing, which may have organically included discussions around structural barriers, stigma, and discrimination related to race, ethnicity, gender or sexual orientation. Non-adherence related to stigma, racial trauma, or discrimination, known to influence HIV outcomes [36], were not specifically targeted as part of the intervention sessions. In future work with EMF, the potential impact of approaches that identify structural level factors influencing individual level behaviors should be explored.

Monitoring devices and apps are a promising technology for dose monitoring and adherence reflection. With the increase in electronic health records and overall health digitization, innovative forms of data presentation and aggregation are possible and can lead to more thoughtful reflection. With the introduction of Long Acting Injectable antiretroviral therapy, youth may have the opportunity to move from daily adherence to an oral regimen to receiving injections at point of care every other- or every 6-months. Visualization of critical outcomes (e.g., viral load over time, attendance to injection visits in specified windows) may still provide valuable reflection opportunities for youth. Our data highlights the potential positive impact of visualization tools for health behaviors that could apply to a variety of other behaviors and outcomes.

## Footnotes

1 35 participants complete both week 4 and week 12 intervention sessions, 1 participant only had a transcript for their week 4 session and another participant only had a transcript for the week 12 session.
